# Descriptive Study to Assess Post-acute COVID-19 Complications in Patients Presenting at a Teaching Hospital in North India

**DOI:** 10.7759/cureus.39510

**Published:** 2023-05-26

**Authors:** Vikas Chandra Vidyarthi, Harish Gupta, Ajay Verma, Abhishek Singh, Satish Kumar, Prem Singh

**Affiliations:** 1 Medicine, King George's Medical University, Lucknow, IND; 2 Respiratory Medicine, King George's Medical University, Lucknow, IND; 3 Cardiology, King George's Medical University, Lucknow, IND; 4 Medicine, Ganesh Shankar Vidyarthi Memorial (GSVM) Medical College, Kanpur, IND

**Keywords:** 2d echo, spirometry, breathlessness, fatigue, sars-cov-2, covid 19

## Abstract

Introduction

Coronavirus disease 2019 (COVID-19) caused by a novel coronavirus named severe acute respiratory syndrome coronavirus 2 (SARS-CoV-2), was first reported at the end of 2019 in Wuhan, Hubei Province, People’s Republic of China, at a cluster of unusual pneumonia patients. The outbreak was declared a Public Health Emergency of International Concern on 30 January 2020 by World Health Organization. We are receiving patients in our OPD (Out Patient Department) with a new set of health complications having been infected with COVID-19. We planned to collect our data and try to find by various statistical methods, quantify the complications, and assess how we can deal with the new set of complications we are witnessing in this post-acute COVID-19 group of patients.

Materials and methods

The study was conducted by enrolling the patients at OPD/IPD (In Patient Department) by conducting a detailed history and clinical examination, routine investigations, 2D echocardiography (2D Echo), and pulmonary function test (PFT). The study assessed the worsening of symptoms, new onset symptoms, or the symptoms that continued even in the post-COVID-19 status as post-COVID-19 sequelae.

Results

Maximum cases were male and most of them were asymptomatic. The most common post-COVID-19 symptom that persisted was fatigue. 2D Echo and spirometry were done and changes were noticed even in those subjects who were asymptomatic.

Conclusion

Since significant findings were seen on clinical evaluation accompanied by 2D Echo and spirometry, it is essential to screen all presumed and microbiologically proven cases for long-term follow-up.

## Introduction

Coronavirus disease 2019 (COVID-19) was first reported at the end of 2019 in Wuhan, Hubei Province, People’s Republic of China, as a cluster of pneumonia patients with an unknown cause. These patients were clustered around the local wholesale seafood market [1].

The disease is of presumably zoonotic origin, and the infectious agent's primary hosts are claimed to be bats and intermediate hosts are pangolins. Person-to-person spread occurs by aerosol, respiratory droplets, and contact transmission [2-3].

Patients present with fever, dry cough, sore throat, headache, myalgia, and fatigue. Some uncommon symptoms include abdominal pain and diarrhea, primarily seen in children and adolescents [4-6].

Severe respiratory illness is seen in around 20% of the patients, and the case fatality ratio is approximately 2.3% [7]. Several complications are known to occur, including acute respiratory failure, acute respiratory distress syndrome (ARDS), acute kidney injury, acute liver injury, and multiple organ dysfunction syndromes. Older age, male sex, obesity, and other medical comorbidities are predictors of severe illness [8-10].

It is caused by a novel coronavirus named severe acute respiratory syndrome coronavirus 2 (SARS-CoV-2) by the International Committee on Taxonomy of Viruses (ICTV). The outbreak is worldwide and was declared a Public Health Emergency of International Concern on 30 January 2020 by World Health Organization [10-12].

Several recent studies have explored the psychological impact of COVID-19 on patients infected with COVID-19, the frontline healthcare staff, and the general public [13-18].

We are receiving patients in our Out Patient Department (OPD) with a new set of health complications having been infected with COVID-19. We planned to collect our data and try to find by various statistical methods, quantify the complications, and assess how we can deal with the new set of complications we are witnessing in this post-COVID-19 group of patients.

## Materials and methods

The study design was a prospective cohort study with a sample size of 100. All the patients were included in the study after getting ethical clearance from Research Cell, King George’s Medical University (KGMU), Lucknow (Ref. code: V-PGTSC-11A/P26). The study was conducted in OPD/IPD (In Patient Department) patients in the Department of Medicine, Department of Respiratory Medicine, Department of Cardiology, King George’s Medical University. Patients were enrolled on OPD/IPD basis. Detailed history and examination were made of all patients in the study and they were examined for routine investigations, 2D echocardiography (2D Echo), and pulmonary function tests (PFT). The study assessed the worsening of symptoms, new onset symptoms, or the symptoms that continued even in the post-COVID-19 status as post-COVID-19 sequelae. The results were supplemented using the Centers for Disease Control and Prevention (CDC) and other post-COVID-19 symptom guidelines in the form of a questionnaire as these guidelines evolved in real-time.

All patients included in the study who were microbiologically proven for COVID-19 or were presumed COVID-19 of all genders, of age >12 years, and who gave written informed consent for the study.

## Results

2D Echo and PFT were done on the patients enrolled in the study of which 23% (n=23) had changes seen in 2D Echo and 31% (n=31) had values outside the normal range in spirometry. In the present study, 100 subjects were enrolled, and the mean age was 42.28 years. Out of 100 patients, three patients were known cases of coronary artery disease and one case had rheumatic heart disease.

**Figure 1:**
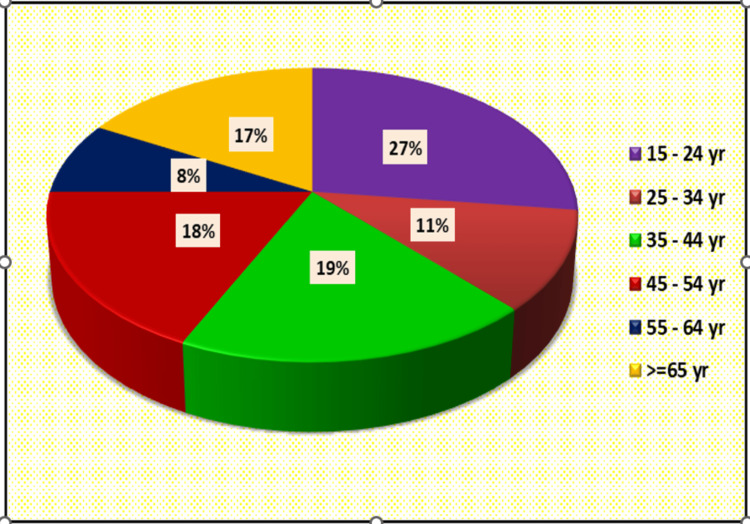
Distribution of cases according to age

**Figure 2:**
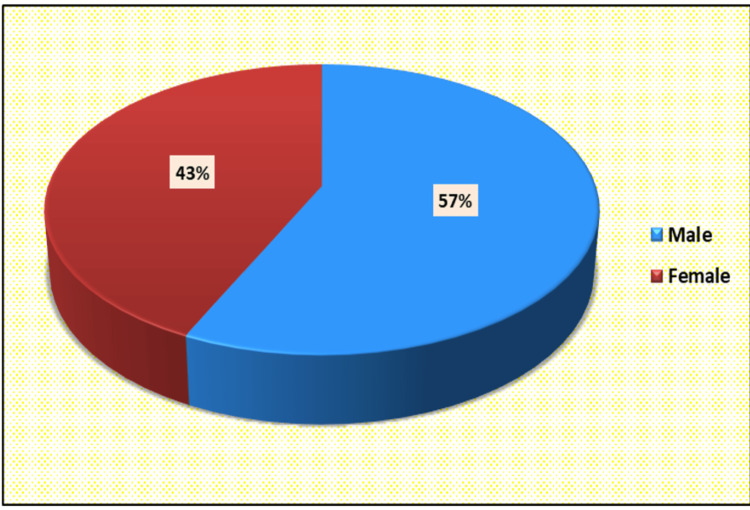
Distributions of cases according to sex

Twenty-seven percent of cases belong to the 15-24 year age group and minimum in the 55-64 age group (n=8) (Figure 1). Maximum cases were male (n=57) (Figure 2).

**Figure 3:**
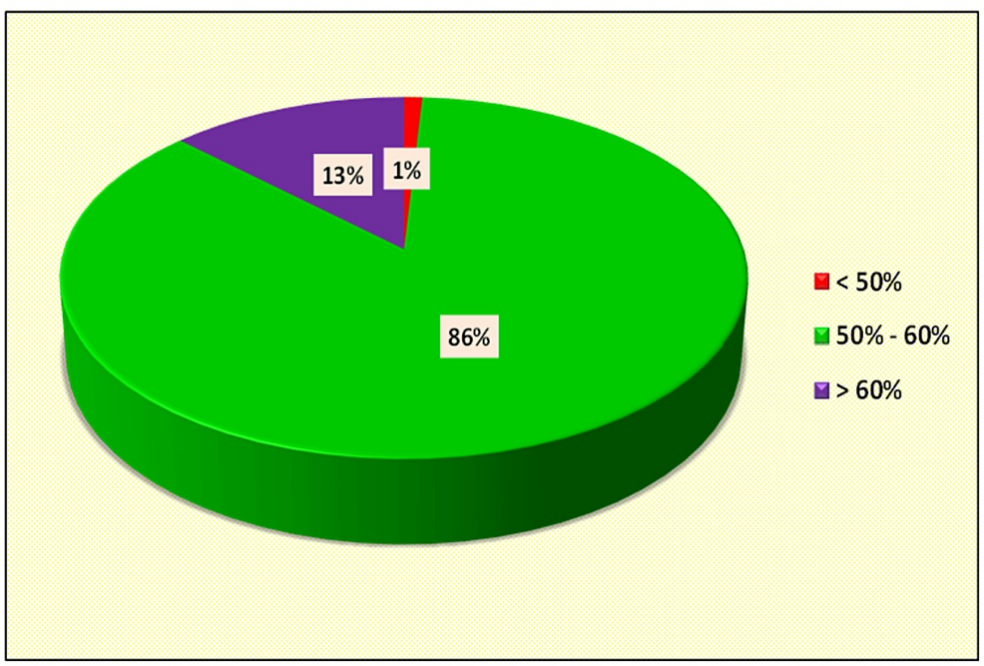
Distribution of cases according to left ventricular ejection fraction

Most of the patients had left ventricular ejection fraction (LVEF) in the range of mid-range ejection fraction i.e: 50-60% and the only patient who had ejection fraction <50% was a known case of ischemic cardiomyopathy (ICMP) (Figure 3). 

**Figure 4:**
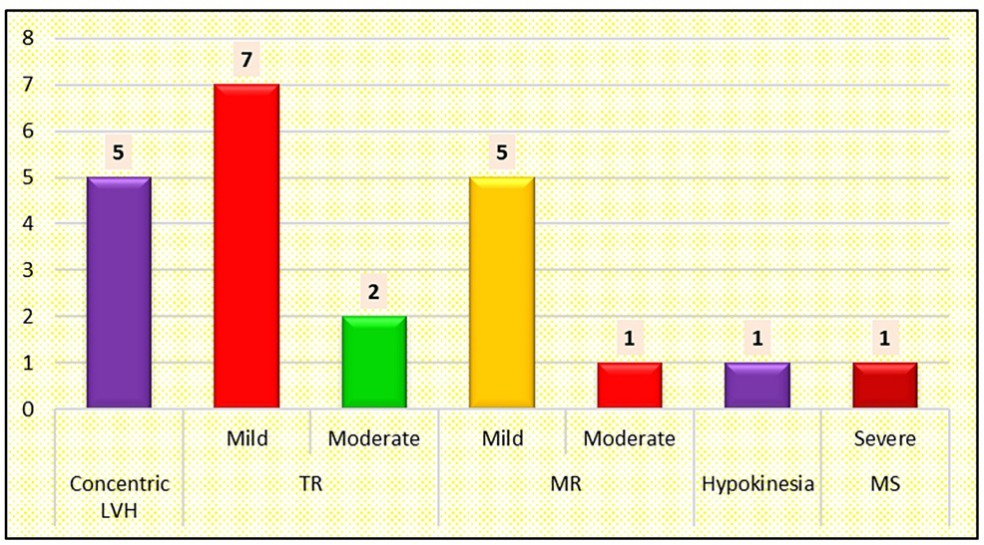
Distribution of Cases according to Cardiac Abnormality LVH, Left Ventricular Hypertrophy; MR, Mitral Regurgitation; TR, Tricuspid Regurgitation; MS, Mitral Stenosis

**Figure 5:**
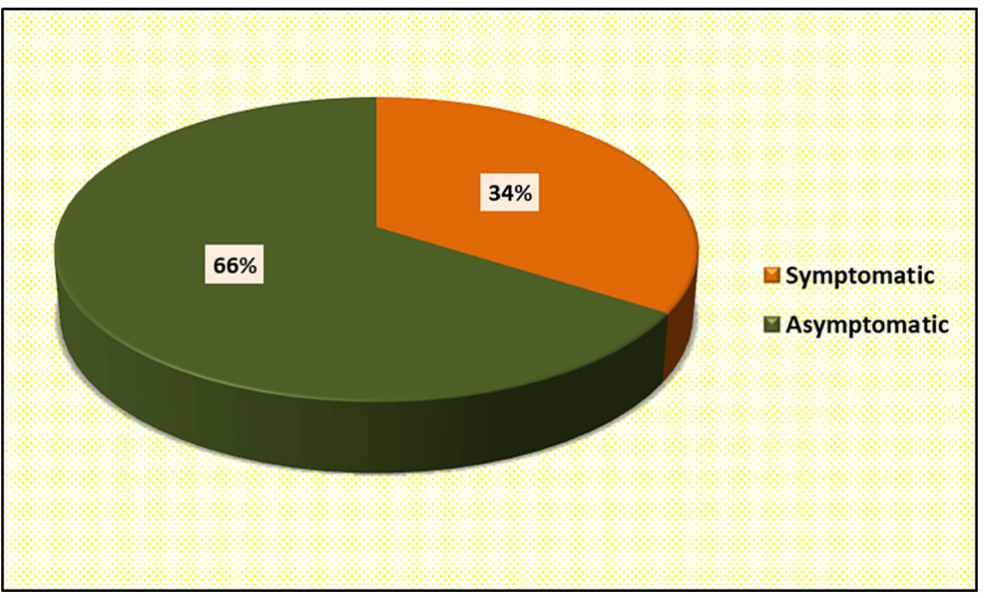
Distribution of cases according to post-COVID-19 type

Concentric left ventricular hypertrophy (LVH) was reported in five subjects, moderate tricuspid regurgitation (TR) in two subjects, moderate mitral regurgitation (MR) in one subject, and severe mitral stenosis (MS) in one subject. Global hypokinesia was reported in one case (Figure 4). The majority of the patients were asymptomatic (Figure 5). 

**Figure 6:**
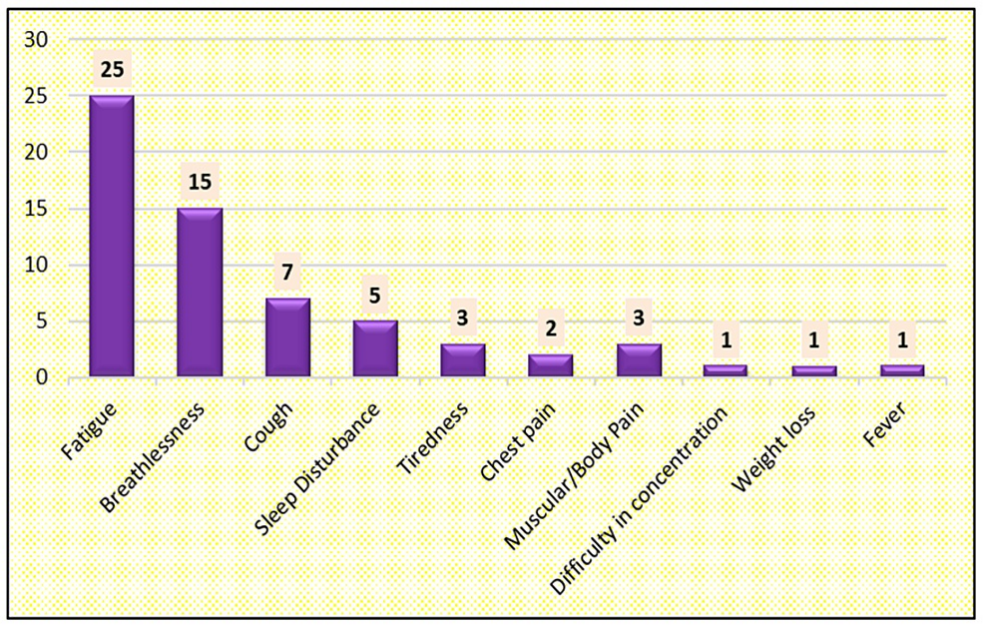
Distribution of cases according to post-acute COVID-19 symptoms

The most common post-acute COVID-19 symptoms that persisted after 12 weeks of discharge were fatigue (n=25), breathlessness (n=15), cough (n=7), sleep disturbance (n=5), tiredness (n=3), chest pain (n=2), muscular pain (n=1), and difficulty in concentration (n=1) (Figure 6). 

In the study, we did the spirometry of the patients enrolled; out of the total enrolled 25% were lost to follow-up/could not do/expired during the study.

**Figure 7:**
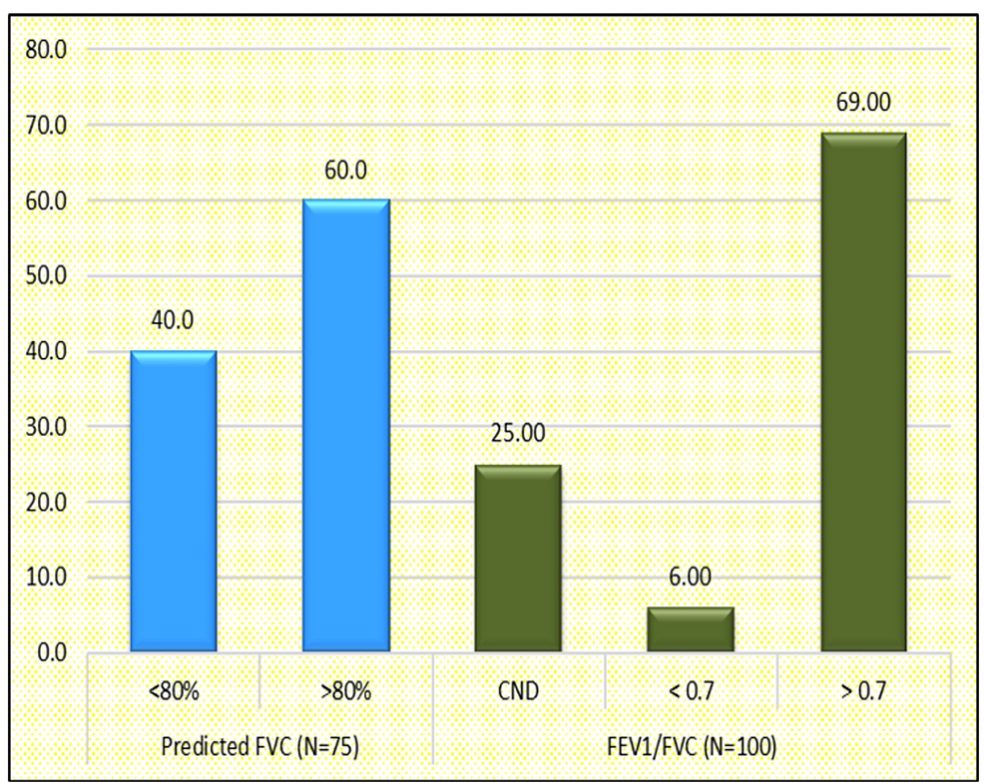
Distribution of cases according to FEV1/FVC FEV1, Forced Expiratory Volume in one second; FVC, Forced Vital Capacity; CND, Could Not do

**Figure 8:**
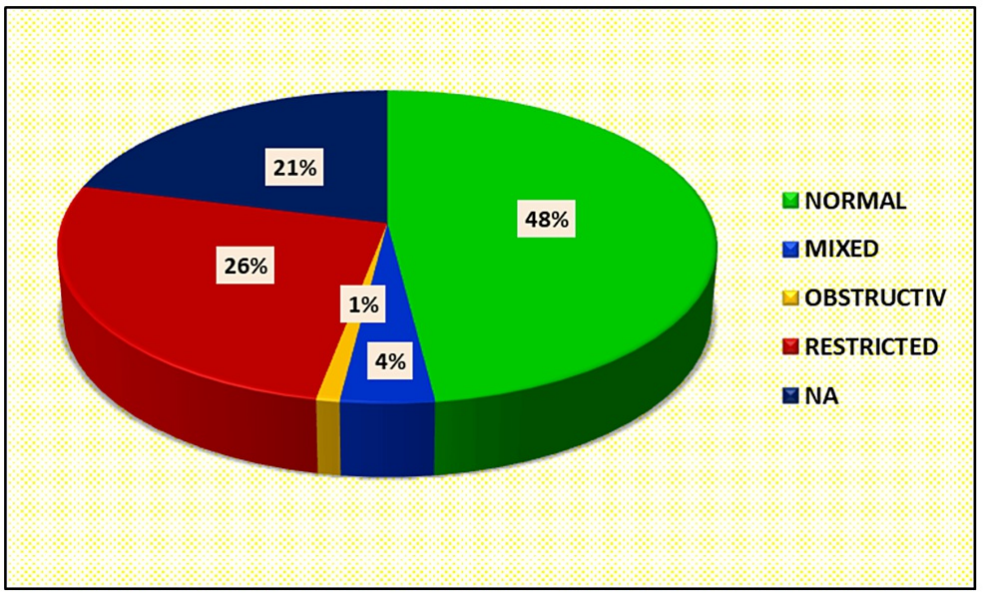
Distribution of cases according to observed pattern on pulmonary function test NA, Not Applicable.

Out of all who could perform the spirometry: a) six patients had forced expiratory volume in one second (FEV1)/forced vital capacity (FVC) <0.7; b) 69 patients had FEV1/FVC >0.7; c) FVC <80% was present in 30 subjects, FVC >80% was present in 45 subjects (Figure 7). Twenty-six patients had a restrictive pattern, one had an obstructive pattern, and four had a mixed pattern (Figure 8). 

## Discussion

The present study aimed to analyze the after-effects of the COVID-19 pandemic on patients presenting to our hospital. The most common post-acute COVID-19 symptom that persisted as post-COVID-19 sequelae was fatigue, while some reported changes in sleeping patterns.

Sonnweber et al. conducted a study in which they conducted a detailed interview, clinical examination, laboratory testing, lung function analysis, and echocardiography and evaluated the cardiopulmonary damage in 145 subjects recovering from COVID-19 at 60 and 100 days after confirmed diagnosis, 41% of all subjects exhibited persistent symptoms 100 days after COVID-19 onset [19], while in our study 34% were symptomatic.

Townsend et al. conducted a study in which 110 COVID-19 patients were evaluated after discharge 90 days from hospitalization due to acute COVID-19; fatigue and dyspnoea (39%) were the most common symptoms noted, followed by sleep disturbance and chest pain [20], while in our study the prevalence of fatigue and dyspnoea (40%) was followed by sleep disturbance and chest pain.

Ngai et al. did a study on post-acute COVID-19 patients and reported 28% of the survivors presented with decreased lung function [21], while in our study 31% (n=31) of the survivors had decreased lung function.

An observational study was conducted by Garcia‑Zamora et al., in which 58.2% were asymptomatic, in contrast, to which we reported 37% of subjects to be asymptomatic. In the same study, the most common post-acute COVID-19 symptom was breathlessness (47.4%), but in our study, the most common and prevalent post-COVID-19 symptom was fatigue (39.68%) [22].

In a study done by Garcia‑Zamora et al., 3.7% of the participants had an LVEF below 50%. Out of 3.7% of patients with reduced LVEF, 50.0% of them denied a relevant medical history and were unaware of this condition while in our study the prevalence of LVEF below 50% was 1%, he was symptomatic having breathlessness and was a follow-up case of ischemic cardiomyopathy (ICMP) [22].

The same study detected regional wall motion abnormality in 4.4% of the participants. After excluding those with a history of ischemic cardiomyopathy or depressed LVEF, 1.1% of the participants presented abnormal wall motions in the left ventricle while in our study excluding ischemic heart disease, there was no abnormal wall motion on 2D Echo. Authors of the same study observed that 8.2% of the participants without prior cardiovascular disease or significant comorbidities had male preponderance and had some echocardiographic abnormality, (right ventricle was dilated in 1.6% of participants, 3.1% had a reduced global longitudinal strain (GLS), and 0.27% had reduced right ventricular (RV) function while in our study 7% of the total patients excluding any comorbidity were found to have some echo abnormality while in our study males were more commonly affected than females.

Naik et al. conducted a prospective study and evaluated a north Indian cohort of 1234 COVID-19 patients and found that 40.1% of patients reported persistent symptoms of clinical remission of the acute disease [23]. While in our study 34% were symptomatic and complained of persistent symptoms as a post-acute COVID-19 sequelae.

Rao et al. did a web-based survey on 2038 respondents with almost 40.1% reporting post-COVID-19 syndrome beyond three months since the acute disease and fatigue was the most common complaint. A study in the Asian Institute of Gastroenterology Hospitals, Hyderabad, India collected data from a younger cohort of patients (N=773, median age 34 years) and reported the prevalence of post-COVID-19 syndrome (against the most common symptom being fatigue) to be 12.8% at three months diagnosis [24]. This study also reported post-COVID-19 syndrome to be more prevalent in females.

Shah et al. conducted a study during the post-acute COVID-19 phase, and reported fatigue as the most common persistent symptom, with 34% experiencing fatigue after 60 days [25] while in our study 25% had persistent complaints of fatigue after 90 days.

Chippa et al. did a study on post-acute COVID-19 syndrome, where he reported that female patients were prone to develop post-acute COVID-19 symptoms, mainly fatigue, anxiety, and depression [26]; this was in concordance with our study i.e. symptomatic females (n=43) and fatigue was the most prevalent post-acute COVID-19 symptom.

Phillips et al. conducted a study on post-acute COVID-19 patients and reported fatigue and breathlessness as the most common post-acute COVID-19 symptom [27], which was in congruence to our study i.e females (n=43) and fatigue being the most prevalent post-acute COVID-19 symptom followed by breathlessness.

Davido et al. did a study in Italy of 143 patients after about a two-week-hospitalization for COVID-19, many patients struggled with symptoms 60 days on average after the onset of their illness; 87% had at least one symptom, particularly fatigue, and dyspnoea [28], which was in congruence to our study i.e. females (n=43) and fatigue being the most prevalent post-acute COVID-19 symptom followed by breathlessness (n=15).

Modi et al. evaluated post-COVID-19 patients on spirometry, where abnormal spirometric parameters were reported in 27 (51.9%) patients, with the restrictive pattern being the most common type of abnormality [29] (n=23, 44.2%).

Patil et al. in a study did the spirometry assessment of post-COVID-19 at 12 weeks post-discharge from the hospital, abnormal lung function in 77.5% of post-acute COVID-19 cases, the restrictive pattern was the predominant type and documented in 43.33% cases, normal lung functions were documented in 22.5% cases [30]. In our study also the restrictive pattern was the most common (n=26, 34.6%).

All the above-mentioned studies performed spirometry analysis in one-month post-discharge. These studies suggest that patients affected by COVID-19 pneumonia are at increased risk of developing restrictive pulmonary diseases after recovery from the acute illness if they survive. Case reports/case series are emerging from several medical centers analysing and documenting several findings that we observed in our study. There may be several explanations for the persistence of symptoms and examined signs in such patients, dysfunction of the cardiopulmonary system, central nervous system (CNS), higher mental functions, persistence of the virus, and dysfunctional immune response; all are possible explanations for this observation. Prospective randomized observational, case-control, cohort study will enhance our understanding of the disease process and possibly may help us in managing such patients.

There were certain limitations while conducting our study as challenges arose while comparing with the evolving literature due to widely differing study methodologies, the small sample size in the present study and the limited availability of comparative studies, differing case definitions of post-acute COVID-19 in the literature, etc.

## Conclusions

Pulmonary function abnormality is common after post-COVID-19 pneumonia cases so should not be overlooked and thoroughly screened for it. Restrictive lung disease is the predominant lung functional impairment in post-COVID-19 cases. Age above 50 years, male gender, high CT severity score, and longer duration of illness, have documented significant impacts on post-COVID-19 lung functions. Timing of spirometry was important in follow-up post-COVID-19 cases, as ongoing inflammation till one month of duration of illness may have a negative impact on the findings of spirometry and can result in false results. For the last three years or so we have been witnessing a pandemic of COVID-19 unfolding in our era. Now medical literature around the world has registered and recorded accounts of the acute presentation of the disease as it swept different geographies in its different waves.

Simultaneously after the beginning of the first wave, we also started to witness post-acute complications as described by patient groups in the early stages. But knowledge about the less -well- recognized illness, as compared to its acute counterpart, is still emerging and we are increasing our wits in real-time as the infectious disease changes into a new form on a 'regular' basis. As cases of the disease present at primary healthcare centers and clinics of family physicians, we present our experience while examining such patients. By regularly updating our pool of case findings earned in this way, it may be possible to manage such patients in a better way. By regularly and continuously spreading case studies of this group of patients, we may enhance, and then capitalize upon, the collective efforts. As the journey against the novel Coronavirus is still on, passing on the information may help our fraternity providing primary care to rapidly detect this disease state and may expedite the process of proper assessment and formal diagnosis.

## References

[1] She J, Jiang J, Ye L, Hu L, Bai C, Song Y (2020). 2019 novel coronavirus of pneumonia in Wuhan, China: emerging attack and management strategies. Clin Transl Med.

[2] Mackenzie JS, Smith DW (2020). COVID-19: a novel zoonotic disease caused by a coronavirus from China: what we know and what we don't. Microbiol Aust.

[3] Machhi J, Herskovitz J, Senan AM (2020). The natural history, pathobiology, and clinical manifestations of SARS-CoV-2 infections. J Neuroimmune Pharmacol.

[4] Baj J, Karakuła-Juchnowicz H, Teresiński G (2020). COVID-19: specific and non-specific clinical manifestations and symptoms: the current state of knowledge. J Clin Med.

[5] Ata F, Almasri H, Sajid J, Yousaf Z (2020). COVID-19 presenting with diarrhoea and hyponatraemia. BMJ Case Rep.

[6] Sharma R, Agarwal M, Gupta M, Somendra S, Saxena SK (2020). Clinical characteristics and differential clinical diagnosis of novel coronavirus disease (COVID-19). Coronavirus Disease 2019 (COVID-19).

[7] Deora S, Bhardwaj P, Garg MK (2020). COVID-19 pandemic: clinical management protocols for cardiac disease patients at teaching institute in Western Rajasthan. J Family Med Prim Care.

[8] Alimohamadi Y, Tola HH, Abbasi-Ghahramanloo A, Janani M, Sepandi M (2021). Case fatality rate of COVID-19: a systematic review and meta-analysis. J Prev Med Hyg.

[9] Dana R, Bannay A, Bourst P (2021). Obesity and mortality in critically ill COVID-19 patients with respiratory failure. Int J Obes (Lond).

[10] van Son J, Oussaada SM, Şekercan A (2021). Overweight and obesity are associated with acute kidney injury and acute respiratory distress syndrome, but not with increased mortality in hospitalized COVID-19 patients: a retrospective cohort study. Front Endocrinol (Lausanne).

[11] Naderi N, Ansari Ramandi MM, Baay M (2020). Cardiovascular patients in COVID-19 era, a case series, an experience from a tertiary cardiovascular center in Tehran, Iran. Clin Case Rep.

[12] Jee Y (2020). WHO International Health Regulations Emergency Committee for the COVID-19 outbreak. Epidemiol Health.

[13] (2020). Note from the editors: World Health Organization declares novel coronavirus (2019-nCoV) sixth public health emergency of international concern. Euro Surveill.

[14] Cucinotta D, Vanelli M (2020). WHO declares COVID-19 a pandemic. Acta Biomed.

[15] Que J, Shi L, Deng J (2020). Psychological impact of the COVID-19 pandemic on healthcare workers: a cross-sectional study in China. Gen Psychiatr.

[16] Hossain MM, Tasnim S, Sultana A (2020). Epidemiology of mental health problems in COVID-19: a review. F1000Res.

[17] Häussl A, Ehmann E, Pacher A (2021). Psychological, physical, and social effects of the COVID-19 pandemic on hospital nurses. Int Nurs Rev.

[18] van der Goot WE, Duvivier RJ, Van Yperen NW (2021). Psychological distress among frontline workers during the COVID-19 pandemic: a mixed-methods study. PLoS One.

[19] Sonnweber T, Sahanic S, Pizzini A (2021). Cardiopulmonary recovery after COVID- 19: an observational prospective multicentre trial. Eur Respir J.

[20] Townsend L, Dyer AH, Jones K (2020). Persistent fatigue following SARS-CoV-2 infection is common and independent of severity of initial infection. PLoS One.

[21] Ngai JC, Ko FW, Ng SS, To KW, Tong M, Hui DS (2010). The long-term impact of severe acute respiratory syndrome on pulmonary function, exercise capacity and health status. Respirology.

[22] Garcia-Zamora S, Picco JM, Lepori AJ (2023). Abnormal echocardiographic findings after COVID-19 infection: a multicenter registry. Int J Cardiovasc Imaging.

[23] Naik S, Haldar SN, Soneja M (2021). Post COVID-19 sequelae: a prospective observational study from Northern India. Drug Discov Ther.

[24] Gella V, Reddy DN, Kulkarni AV, Kumar J, Radhakrishnan M, Chatterjee R, Guduru VR (2022). Postcoronavirus disease-2019 symptoms are not uncommon among recovered patients: a cross-sectional online survey among the Indian population. Lung India.

[25] Shah S, Bhattarai SR, Basnet K (2022). Post-COVID syndrome: a prospective study in a tertiary hospital of Nepal. PLoS One.

[26] Chippa V, Aleem A, Anjum F (2022). Post acute coronavirus (COVID-19) syndrome. StatPearls.

[27] Phillips M, Turner-Stokes L, Wade D (2020). Rehabilitation in the wake of Covid-19 - a phoenix from the ashes. British Society of Rehabilitation Medicine.

[28] Davido B, Seang S, Tubiana R, de Truchis P (2020). Post-COVID-19 chronic symptoms: a postinfectious entity?. Clin Microbiol Infect.

[29] Modi P, Kulkarni S, Nair G (2021). Lung function indices in patients undergoing post-COVID assessment- an observational study. Eur Respir J.

[30] Shital P, Dhumal U, Acharya A (2021). Role of spirometry in lung function assessment in post COVID-19 pneumonia cases: correlation with CT severity, duration of illness, oxygen saturation and ventilatory support in critical care setting in tertiary care setting in India. Saudi J Med.

